# A Framework for Automated Acquisition and Processing of As-Built Data with Autonomous Unmanned Aerial Vehicles †

**DOI:** 10.3390/s19204513

**Published:** 2019-10-17

**Authors:** Henk Freimuth, Markus König

**Affiliations:** Chair of Computing in Engineering, Ruhr-Universität Bochum, 44787 Bochum, Germany

**Keywords:** as-built, data acquisition, BIM, UAV, point cloud, octree, ROS, depth camera, obstacle avoidance

## Abstract

Planning and scheduling in construction heavily depend on current information about the state of construction processes. However, the acquisition process for visual data requires human personnel to take photographs of construction objects. We propose using unmanned aerial vehicle (UAVs) for automated creation of images and point cloud data of particular construction objects. The method extracts locations of objects that require inspection from Four Dimensional Building Information Modelling (4D-BIM). With this information at hand viable flight missions around the known structures of the construction site are computed. During flight, the UAV uses stereo cameras to detect and avoid any obstacles that are not known to the model, for example moving humans or machinery. The combination of pre-computed waypoint missions and reactive avoidance ensures deterministic routing from takeoff to landing and operational safety for humans and machines. During flight, an additional software component compares the captured point cloud data with the model data, enabling automatic per-object completion checking or reconstruction. The prototype is developed in the Robot Operating System (ROS) and evaluated in Software-In-The-Loop (SITL) simulations for the sake of being executable on real UAVs.

## 1. Introduction

Large above-ground construction projects require construction management staff to efficiently organise the work of many different contractors. The emergence of Building Information Modelling (BIM) provided all parties involved with a standardised, unified way of managing building-related information 4D-BIM, an extension to BIM that also accounts for change over time, enables management staff to create and maintain schedules of all construction processes directly on the model data. The advantage of this approach is that modifications can be made on a model that includes information of all contractors. Conflicts, whether they are of organisational, spatial or technical nature, can be avoided, because all relevant information is in one place. However, during construction, changes to processes can occur and the model must stay updated at all times. Each process deviation, for example delivery delays, must be accounted for in the model, because as soon as the model does not reflect the actual situation on site, no valuable decisions can be made based on the model’s information. Generation and integration of on-site information in BIM are commonly referred to as scan-to-BIM or as-built [1,2]. A traditional method of producing as-built information is for personnel to go on inspection rounds with printed check-lists and taking photographs for documentation. Afterwards, the newly gathered information needs to be put into files and models and transferred to contractors and all relevant staff. In other cases, contractors may communicate changes to management staff as they occur but still, information must be communicated and the model needs to be updated manually. The efforts of maintaining an updated model and schedule become increasingly complex with construction projects that are large in spatial extent as well as in organisational terms.

As-built generation implies several different types of information. Many types of as-built information are expressed in numbers or dates and are therefore of an organisational nature. This includes material volumes and dates of process events (e.g., delivery dates) which must be tracked to ensure a meaningful state of the planning model. Geometric information is required for avoiding clashes of different objects, for example structural elements must preserve spaces for pipes or general spatial constraints must be satisfied for mechanical, electrical and plumbing (MEP) works [3]. A valuable as-built method should produce visual representations of physical objects, as these are fastest to evaluate for a human viewer. But more importantly, as-built artefacts should be analysable and structured for purposes of automated processing and comparison operations with as-planned model data. This requires as-built data to be segmented in semantic objects, compatible with the object structure of as-planned BIM. Point clouds are the common data structure for scans of physical objects. In practice, point clouds are generated either with Terrestrial Laser Scanning (TLS) devices or image-based photogrammetry. Point clouds may be visualised for intuitive interpretation by the human eye but they are also a suitable source of information for structured analysis. Each point has a geometric location that may be attributed to model space given a correct registration of the point cloud. While the process of creating a laser scan of a certain space is a straightforward task, the structured, automatic creation of point clouds that cover relevant object surfaces involves substation manual labour and expertise. While technical requirements increased in comparison to manual inspection with photos and check-lists, modern as-built methods must also be more efficient in operational terms. Increased construction complexity means more processes that need to be controlled. Furthermore, the rate of producing as-built information must be increased from weekly inspections towards a workflow that maintains correct information on a daily basis.

\ifAC@uppercase@first\expandafter\MakeUppercase\fi 3 (2s), also referred to as drones, are expected to be of great value for problems involving remote sensing in hard to reach locations and automation. The term UAV applies to all types of unmanned aircraft, including plane-type aircraft with wings, multirotor aircraft, palm-sized helicopters and blimps. The preferable form factor in this context are multirotor UAVs. Their aerodynamic characteristics include vertical takeoff and landing, stable hover and the ability to carry payloads like cameras and dedicated axis stabilisation gimbals. In civil engineering, research on UAVs is targeted in different directions such as deterioration analysis in structural health monitoring (SHM) [4], quality control [5,6] and digital reconstruction [7]. Effectively, UAVs are a utility for placing exchangeable sensors in remote locations. The most advantageous of property of these devices lies in the potential of autonomous operation. Autonomy in robots denotes a breaking point for tasks that could not be automated before. Whereas every single acquisition of an as-built artefact involves human effort, an autonomous agent can solve this task repeatedly at no additional cost. For these reasons UAV technology should be investigated as the basis for an efficient as-built method. While most applications focus on the ability to take measurements from remote location and rely on human piloting, only few applications have made practical use of autonomy. Autonomy in this context is the ability of a robotic agent to proactively follow a path towards a target location while avoiding collision with physical objects on the way. This is the minimum requirement that must be satisfied to effectively reduce the effort required by a UAV-based replacement for traditional sensing tasks.

Recent, publicly available work on the PX4 project in combination with the Robot Operating System (ROS) reveals a robust foundation for applications of autonomous UAVs. The autonomous flight control software, which is used in the prototype made for this paper, is proven to work on real UAVs and encourages a development workflow with simulated tests prior to outdoor flight tests.

PX4 is part of an open source robotics ecosystem with contributions from researchers, robotics companies and enthusiasts. Apart from software, PX4 has already published several iterations of the Pixhawk flight control, a reference design for flight control devices which is widespread across commercial products and research UAVs. The framework presented in this work proposes a combination of openly available robotics software and hardware, off-the-shelf stereo cameras as main sensor and BIM data as a source for path generation and processing of as-built data. A software prototype in the form of ROS packages was developed to demonstrate a fully automatic workflow from controlling the UAV to analysing point clouds with the intention of matching scanned objects with their BIM counterparts. Prior work in this ongoing project focused on pre-computing flight missions for UAVs with the goal of taking photos of building facades for inspection purposes. The concept was limited by only relying on global positioning system (GPS) for localisation of the aircraft, which is unreliable in the vicinity of large building structures.

### Contribution

This work proposes a framework for automated as-built data generation with autonomous UAVs. A prototype of the framework was implemented in a toolchain which makes use of existing robotics tools and adds new software to facilitate the automatic robot. The prototype demonstrates the feasibility of the concept and allows a practical evaluation. This work is a continuation of an ongoing research effort to employ UAVs for for inspection and monitoring purposes in construction [8]. The underlying autopilot and robotics software are under active development. Earlier works demonstrated practical limitations due to inaccurate localisation near buildings and unreliable collision avoidance [9]. The framework overcomes these limitations by employing stereo cameras and point clouds for environment perception, localisation and as-built generation. The concept continues to generate targeted flight missions towards individual objects. The known environment is sourced from as-planned BIM, assuming that a 4D-BIM provides a sufficiently complete representation of the construction site environment for the time of the scanning procedure. Since the UAV produces as-built data itself, objects that were detected in earlier flights but missing in the as-planned model, can also be accounted for in subsequent flight missions. The vision-based environment sensor complements the concept, allowing the UAV to actively avoid any physical object unknown at the mission generation stage. Furthermore, the improved localisation of the UAV enables real time registration of the point clouds for processing on the model data.

Apart from automatic acquisition of visual data, the framework defines a method for processing point clouds and a segmentation process for object detection. The processing toolchain compares the geometric representation of single as-planned objects with the input point clouds and segments the unordered data. The object-point clouds are then used to analyse to what extent the input data matches the as-planned object. This matching process enables an automatic identification of single as-built objects and process finalisation checks.

## 2. State of Technology and Research

As-built data and reconstructed 3D geometry in general are valuable assets, which are used for progress tracking [1,3,10], structural health monitoring [11,12], quality assessment [13,14,15] and as-built modelling [16]. As-built information may also be used for increasing construction safety. Alizadehsalehi et al. [17] conducted a study about the potential benefits of UAVs-based inspection for the identification of hazardous zones. The proposed method implies UAVs as a tool to highlight hazards in 4D-BIM models. Each of these tasks relies on efficient data generation methods as it can only be conducted with current and accurate information at hand. Huber et al. [18] describe the process of creating as-built data as time-consuming and error-prone, making it one of the key barriers to the widespread use of as-built BIMs in industry.

One method for acquiring geometric as-built information is to apply photogrammetric methods on photos. Documenting and communicating the state of objects with photos is ubiquitous and experienced human viewers can intuitively recognise important features within an instant after looking at a picture. Structured analysis and photogrammetry for as-built BIM however present challenges towards the way photos are taken.

One approach is to take photos of objects and register them by estimating the camera pose, with which the image was taken. A virtual camera in the model representation is given the reconstructed pose of the photo. The image rendered from the virtual camera’s perspective is comparable to the real photo and differences between the two may be extracted automatically [19,20,21]. Studies based on this approach show qualitative statements about surface properties, spatial objects (also BIM elements) and potential clashes.

The more common approach of producing as-built information with images is to apply structure from motion (SfM) to derive 3D geometry in the form of dense point clouds. In order to achieve an adequate point density, multiple overlapping photos must be taken for every feature of an object that is relevant. The process is computationally intensive but the availability of streamlined software applications has made the process widely available. Golparvar-Fard Mani et al. [22] present an approach where feature points in the reconstructed point cloud are used to mark voxels in the as-planned model as occupied or free. The discretised voxels mark spaces of objects where a physical occupancy was detected and therefore, leading to an automated progress monitoring. Klein et al. [11] conducted a study on generating exterior and interior as-built models with photogrammetry methods. The authors acknowledge limitations regarding measurements of outer structures in upper stories, a limitation that could be mitigated by using UAVs for image acquisition.

UAVs can significantly improve the image acquisition process common in these approaches. Especially the camera registration, required in all works, can be solved by using a UAV that already constantly localises itself for navigation purposes. The requirement for repeatedly taking photos from identical perspectives also becomes effortless with a UAV that may be sent to take a photo from the same position every day.

Besada et al. [23] propose a UAV mission generation approach for inspection purposes. The application allows surveyors to place geometric shapes on objects represented on a 3D map and creates waypoint missions for UAVs along the surface of the geometric shape.

Braun and Borrmann [24] propose a method for automated progress monitoring based on aerial images captured by a UAV with an emphasis on detecting BIM elements in 2D images. A Convolutional Neural Network (CNN) was used for detection and classification of BIM elements in images with the goal of creating a method for automatically checking individual elements of as-planned processes. The tedious and also automation inhibiting aspect of creating training datasets was bypassed by generating labelled data from aerial images automatically. By projecting BIM elements onto images and generating labelled datasets from those parts of images that lie within the projected area, no manual cropping and labelling were necessary. The camera poses required for the projection step were derived from photogrammetric camera pose estimation. The achievements of this work could benefit from introducing automation to the process of capturing visual data with autonomous UAVs. Furthermore, a deterministic approach to guiding an autonomous UAV around the construction site could systematically avoid some types of occlusions, a problem also acknowledged by the authors.

Reconstruction of geometry with Terrestrial Laser Scanning (TLS) is a common method that is generally more straightforward and accurate than photogrammetry. The method, however, has other drawbacks compared to image-based approaches. TLS devices require steady placement during scan time which introduces a setup time before each scanning operation. The scanning process itself consumes further time, during which no work can be done in the scanning area. Conventional TLS only reconstructs geometry, whereas photogrammetry also preserves colour values to be mapped on each point in the resulting point cloud. Furthermore, reconstruction of objects with surfaces oriented in multiple directions requires multiple scans which introduces registration tasks for each scan [16]. Reconstruction of a single object may be more accurate with TLS but the implied effort is not agreeable with as-built applications, where multiple reconstructions are required on a daily basis.

In conclusion, the two presented methods for geometry reconstruction complement each other. With photogrammetry, ease of use introduces a drawback in accuracy, whereas TLS requires extensive efforts and provides higher accuracy. Setup and operation of TLS-based inspections are time-consuming and cause interferences with other construction tasks [16]. Considering, that as-built tasks aim to make construction management more efficient, image-based methods are preferred.

Point cloud data, regardless of the acquisition method, has no inherent alignment in the model space. Point clouds derived from images have no accurate position information, eventually present GPS information provided by some cameras is neglected in this case for reasons of accuracy and sensitivity to disturbances. Another approach to point cloud registration implies the placement of markers on scanned objects. While markers simplify a single registration process, the method would require visible placement of markers for every structural element, an impractical effort that thwarts the automation requirement. Mentasti and Pedersini [25] propose a method for scanning large objects with UAVs based on physical markers that are being laid out around each target object. Automatic registration approaches imply techniques like Iterative Closest Point (ICP) or the 4-Points Congruent Sets method [26,27,28] and an adaptation for registration of point clouds on planes of meshed model geometry. The registration of point clouds with different geometric representations such as meshes, found in BIM models, poses special problems. Bueno et al. [29] adapted the 4-Points Congruent Sets method as 4-Plane Congruent Set algorithm with robust results albeit being computationally expensive.

Park et al. [30] propose a method for registration of point clouds with UAVs and unmanned ground vehicle (UGVs) for extended visual coverage. The authors made use of real-time kinematic (RTK) technology to improve localisation to an expected 2.5 cm. While RTK significantly improves localisation accuracy, it is still susceptible to multipathing effects and other perception issues like all common global navigation satellite system (GNSS) methods. Furthermore, RTK does not improve on reliability and safe operation since there is no means of perceiving objects in the environment.

After acquisition and registration of geometry information, the point cloud is still unstructured in comparison to the target BIM. At this stage, only a human viewer can intuitively attribute points to different objects. A segmentation is therefore required that separates point clouds into groups of single, smaller point clouds that describe individual objects. Approaches include colour-based segmentation with region growing [31] or shape/plane detection [32,33].

### 2.1. Choosing a Suitable Platform for UAV Development

Employing UAVs in the context of an application like as-built BIM poses special requirements towards interoperability of the device. Common commercial UAVs support following GPS waypoints and offer basic mission flight modes. Mission flight modes are limited to the extent that waypoints can only be set manually through a smartphone application or a comparable interface that is integrated within the remote control. Some manufacturers offer optional development programs in the form of Software Development Kit (SDKs). While SDKs provide limited access for integrating commercial UAVs within specialised applications, the commitment to a proprietary interface implies an unnecessary dependency towards the company to maintain and support the hardware and software. Open robotics platforms fill this gap by adhering to open standards using established tools.

PX4 is an open source software and hardware platform for UAVs. It was initially founded as a research platform for vision-based autonomy and became a widely adopted UAV platform. PX4 provides full control over the UAV and extensive compatibility with the Robot Operating System (ROS). Developers and testers provides feedback and analysable flight logs before proposed changes are accepted for roll-out to users in releases. This denotes a significant advantage in quality control over many other platforms.

The project PX4/avoidance is a further basis of this work. It is focused on reactive autonomy and obstacle avoidance with path planning in known and unknown environments. The avoidance technique is regularly evaluated in simulated and real flight tests [34].

ROS is an open source pseudo operating system for the development of and execution of robotics software on robot hardware. At the core of the ROS concept is a graph architecture, which considers all robot-related processes and entities as distinct nodes while messages passed among nodes are considered edges. Nodes are executed as independent system processes on the host operating system and adhere to the ROS interface. Apart from the architectural aspect, ROS is also a large collection of established robotics modules that come from an international community. Abstraction of message types and networking communication allows single modules of robotic systems to be executed on different computer systems. Furthermore, the modular architecture makes nodes exchangeable under the premise of conformity with the required message protocol. This is an essential aspect for research purposes, as it allows developers to change certain functionality of a robot with little effort.

Hrabia et al. [35] conducted a case study on supporting firefighters with UAVs for safety management in emergency situations. The authors chose ROS and PX4 as the basis for a UAV system that implements high-level mission planning, Simultaneous Localisation and Mapping (SLAM) on stereo images and object recognition based on CNN.

Iterative changes in code need testing and verification but conducting outdoor experiments for every test is impracticable. Malfunctioning code could destroy hardware or even put humans in danger when testing flying robots. Therefore, simulation driven development and testing have become indispensable utilities.

### 2.2. Simulation in Robotics

This work substantially builds on the premise that the simulation of the robotic system, implemented in ROS, is realistic enough to indicate that the same behaviour of the robot can be expected when executed on the real aircraft. Simulation does not completely replace real flight tests, which will be subject of further studies but is considered a practical tool during prototyping stages.

Simulation is a key method for the development of robots, especially with autonomous UAVs, where malfunction on the device causes crashes, destroys hardware and possibly causes harm to bystanders and objects. Outdoor experiments with untested features significantly slow down the development process and pose unwanted risks and costs. For these reasons a robotics development workflow emerged that employs realistic simulations up to a point of validation where real world experiments can be conducted [36,37,38].

Gazebo is a popular simulator with deep integration in ROS [39,40]. Koenig and Howard [39] present an overview of the design and usage concepts that went into the creation of Gazebo. While the application has evolved substantially since the initial publication, the core principles still hold up. At its core is the world model, a hierarchical representation of user-defined robot environments. The simulator can be configured to use exchangeable physics engines and a plugin system enables integration of new virtualised sensors, such as virtual cameras.

When used for UAV development, the control software of the UAV is executed on the development machine (SITL) and its input/output is coupled to Gazebo instead of real sensors and actuators. In brief, the procedure can be summarised as follows: If the UAV-instance outputs a motor signal, the propellers of the simulated vehicle will start spinning. According to the physical description of the vehicle, the spinning propeller will generate thrust and autorotation. The physics engine processes the thrust and eventually the vehicle will lift off the ground as soon as the generated thrust is greater than the gravitational force which accelerates the vehicle in the opposite direction. At the same time, forces affecting the vehicle are registered by the UAV’s inertial measurement unit (IMU), where the feedback loop will compute the next set of motor signals for stabilisation. Given an accurate model of physical properties and effects, the same setup will behave virtually identical in the real world.

The real benefit here is that experiments carried out in the simulation run the same code as the real UAV in outdoor usage. When transitioning from simulator experiments to outdoor execution, only the virtual sensors need to be replaced by their real counterparts. As shown in the works above, this type of simulation is proven to be transferable to the real world.

Hardware-In-The-Loop (HITL) is a similar approach to SITL that uses the actual autopilot device to execute on the simulated data. HITL is more interesting for performance-related tasks, where the actual timings of the microcontroller are required. [41] used a manually controlled, HITL-simulated PX4 UAV for a study on detecting MEP-work (pipes) with CNN.

## 3. Concept and Prototype

The objective of this paper is the development of a novel method for measuring the as-built status with autonomous UAVs. The integration of UAVs requires a technological bridge between model information and controlling the autonomous aircraft. Control in this context means being able to interface with the path planning unit of the UAV’s flight control. The UAV considered for this work is able to autonomously position itself along commanded waypoints. Waypoints are defined as geographic locations with an altitude determined either relative to sea level or relative to the takeoff altitude. This functionality is commonly known as waypoint navigation and typically aided by a GPS sensor. The UAV considered for this work is based on state-of-the-art technology that enables the vehicle to sense objects in its environment and avoid collisions while reaching the next target location. The collision avoidance is based on point clouds derived from depth camera systems. These point clouds are also used for reconstruction and analysis of the as-built status. In order to achieve the expected degree of automation, the UAV must be given correct target locations of construction objects and furthermore knowledge of all known structures from the BIM. The following sections present all relevant components that constitute this automatic system.

### 3.1. Simulating Automated As-Built Inspections with UAVs

Simulation is an important utility in this work. It enables full control over tests and input data, which is not feasible in outdoor tests. Point clouds, the main information input, are created in real time from camera images on synthetic data. Gazebo provides a stereo camera plugin for streaming images from the UAV’s point of view. Simulated cameras are defined by various properties such as resolution, field of view, frame rate, distortion and noise. These parameters make the virtual cameras configurable to resemble the properties of the real camera used on the robot. This allows working with synthetic visual sensors as input for robot perception. Since navigation and as-built generation are based on visual information, the visual preparation of the virtual as-built scenes is a crucial task. As-built scenes are defined in world files which describe the composition and properties of physical objects including the ground profile, building structures, the UAV and additional objects like machinery.

**Figure 1:**
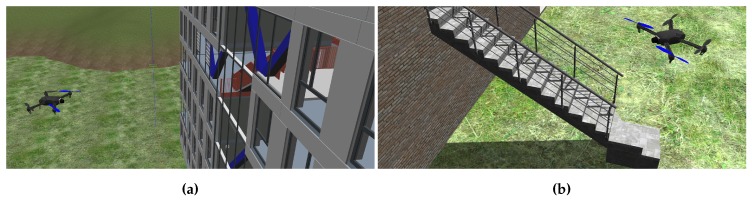
The simulated unmanned aerial vehicle (UAV) autonomously navigates around virtual Building Information Modelling (BIM)-derived building scenes. (**a**) Example of a building model with few surface details. (**b**) Example of a building with realistic textures. The additional surface features greatly improve depth sensing in simulation.

Figure 1 shows two examples of a simulated UAV navigating in synthetic as-built scenes. The aircraft is controlled by the actual autopilot and actively avoiding collisions by visual environment perception. Working with synthetic data allows developers to consciously create situations where as-built deviates from as-planned by modifying the BIM that is the as-built source. BIMs are sourced from the Industry Foundation Classes (IFC) exchange format. IFC implements the standardised information exchange concept of BIM and represents the as-planned state that is used in construction projects. In order to create a synthetic as-built scene, the geometry information in the IFC file is converted to a Collada 3D asset file. Collada is a modern and open 3D asset exchange format which is maintained by the Khronos Group. The conversion is achieved with a tool based on xBim [42]. The definition of simulation scenes is straightforward, however one additional step was found necessary in the preparation of experiments. In the first tests of the vision-based navigation on synthetic data, the UAV started behaving erratically as soon as its cameras were oriented towards the building in the as-built scene. The converted building models were found to expose only few visible surface features. This is due to the fact, that visual material representations of surfaces in BIM models are only described by colours on plain surfaces. The generation of depth maps for navigation greatly depends on distinct surface features. Figure 1a shows an example of such plain surfaces with little distinct properties. Figure 1b shows a modified as-built representation on which building surfaces were given additional photo-realistic brick and concrete textures. After adding realistic surface representations to the as-built structures, the vision-based navigation was restored, also clearly comprehensible in the number of depth pixels in the resulting depth maps of the stereo camera component.

The mission flight mode of PX4 is capable of cooperating with an obstacle avoidance mechanism. The path planner node in ROS is responsible for analysing the environment and computing paths towards singular target points. There are two instances of planning nodes, the local_planner and global_planner. The local planner is able to navigate in a previously unknown environment and find ways around obstacles that come up during the course towards a waypoint. The local planner leverages a key data structure to maintain the current representation of free and blocked directions. The 3DVFH+ [43] is a computationally efficient two-dimensional array of potential motion directions that are either free or blocked. The elements of the array are a projection of a sphere around the UAV described in polar coordinates and therefore represent 3D motion vectors in all directions. The cell’s values are updated in real-time by mapping the distance of the UAV to the next object to a value in the 3DVFH+ that represents the respective flight direction. The global_planner additionally recognises a previously known environment and maintains a global representation of the explored and known environment in a navigation space representation. Both variants of the planner continuously exchange messages with the autopilot through the mavros interface. This allows a separation of PX4 autopilot tasks that typically run on micro-controller devices and more intensive computations in the ROS which can be executed on an on-board companion computer.

### 3.2. Components of the Toolchain

The prototype implementation of the presented framework is a fully functional ROS project. The modular architecture of ROS and the data flow from camera to as-built information makes it behave like a toolchain with subsequent stages of data processing. The toolchain makes use of existing structures from the PX4/avoidance project with additional modules developed specifically for the purpose of measuring the as-built status of construction sites and BIM.

**Figure 2:**
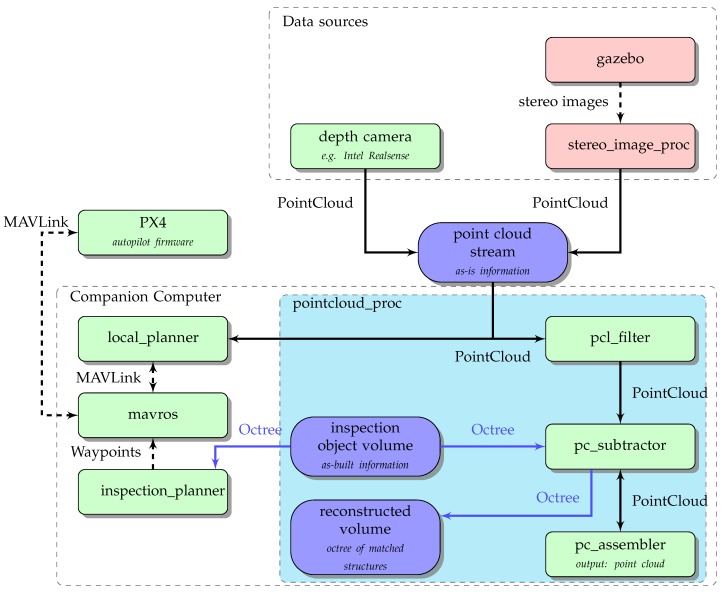
The component architecture of the proposed toolchain. Green nodes represent individual software modules, red nodes indicate simulation components and data artefacts are represented by blue nodes. Solid black arrows represent point cloud data, blue arrows represent access on octrees, any other data is represented by dashed arrows.

Figure 2 provides an overview of the toolchain with an emphasis on the data flow between its modules. Each node in the graph is a software component that handles a certain task and will be presented individually in the following subsections. The relations between nodes denote data exchange and resemble dependencies. The top group labelled Data sources includes nodes that generate the required stream of point cloud data. This stream either comes from stereo cameras on the UAV with integrated depth-image functionality (e.g., Intel RealSense) or from virtual cameras that emulate the real device by capturing images with comparable properties in the 3D simulation environment. The point cloud stream (upper blue node) is needed for obstacle avoidance and for reconstruction of as-built structures, illustrated by the outgoing split data flow.

The stereo_image_proc node (included in recent ROS distributions) is responsible for processing the stereo image stream of the simulated 3D environment.

**Figure 3:**
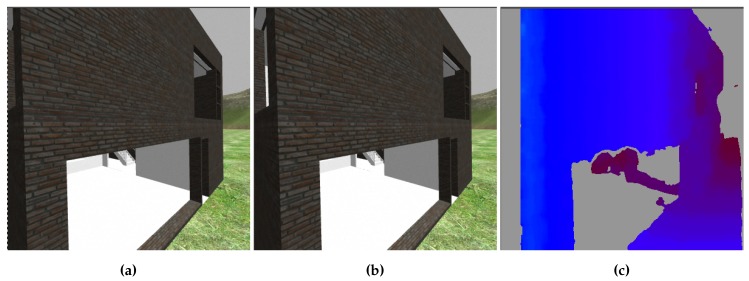
A visualisation of the generation of the disparity map. (**a**,**b**) Images from cameras mounted next to each other. (**c**) The resulting disparity map with a colour gradient indicating perceived depth.

It computes a disparity image, in which each pixel value represents the depth of the same pixel in the rectified input image. Crucial to its ability to derive depth information from stereo images is the semi-global matching (SGM) which identifies matching parts of objects in each pair of images [44]. Figure 3 shows an example of the binocular camera image and the resulting colour-coded disparity map with a gradient between blue and red resembling distances near and far. With depth information and colour available, the node is further capable of producing a ROS typed PointCloud2 message. PointCloud2 is the default type for all further message passing of point cloud data and is compatible with the pcl::PointCloud data structure from the PointCloud Library which offers comprehensive point cloud processing methods, essential to the toolchain’s further operations. The flow of the resulting point cloud data splits at this point, as it is being used as input stream for both the collision avoidance system and the nodes in the subgroup pointcloud_proc. Devices like Intel RealSense can compute disparity maps on their own. The accuracy and performance of an integrated depth camera will be evaluated in upcoming work but is expected to make the processing chain more CPU-friendly. Furthermore, real objects should yield more complete disparity maps than those based on synthetic images which expose fewer visual features.

The subgroup pointcloud_proc encapsulates software modules for real-time processing of point cloud streams and analysis in the octree-based navigation space.

**Figure 4:**
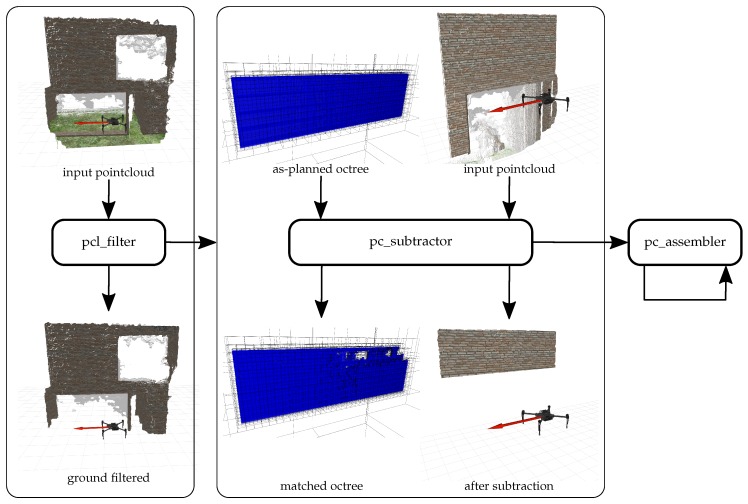
Components of the point cloud processing subgroup. The pcl_filter removes all points at ground-level. The pc_subtractor processes the incoming point cloud with the as-planned octree from the BIM. It generates a matched octree with with occupied cells where physical surfaces were detected. The second output is the object point cloud that represents the target object. The pc_assembler optionally collects all subtracted point clouds and accumulates them for complete coverage.

See Figure 4 for a detailed illustration of the components. pcl_filter is the first node in the subgroup to process the incoming point cloud stream. It is a Point Cloud Library (PCL)-based pass-through filter for eliminating all points outside a certain spatial range in one dimension. In this case, the pass-through filter is used to eliminate all points near ground by discarding all points within a certain margin around takeoff altitude. Figure 4 shows an input point cloud with green patches from the simulation environment. After the passing the filter, the point cloud only contains points of the building structure. The purpose of this filter is not to work perfectly accurate but to eliminate a large share of points that need not be processed in subsequent processes at little computational cost. Ground points are relevant to the obstacle avoidance but since the objective of this toolchain is the measurement of building structures, ground points are of no further interest in the following processing steps. The filter operation increases the performance of all subsequent operations. PCL implements further filtering routines. The statistical outlier removal is one notable filter by which point clouds can be freed from outlier points that do not represent solid structures. During evaluation the result were significantly improved by integrating the statistical outlier removal. See Section 4 for discussion and observations. After pre-processing and filtering, the point cloud data is passed to the subtractor node (pc_subtractor).

### 3.3. The Point Cloud Subtractor

The point cloud subtractor (pc_subtractor) is the second stage in the pointcloud_proc subgroup and implements the key functionality of the toolchain. It reduces and separates input point clouds into groups of points that belong to distinct inspection objects. The node constantly analyses the ratio of matched parts of the as-planned status and produces point clouds that describe individual objects. This enables matching point clouds of construction objects with as-planned model data and thereby drawing conclusions about diverging as-built objects.

The point cloud subtractor requires a spatial description of as-planned object. Point locations in point clouds, especially if captured from a flying vehicle, have a certain error. Two points in slightly different locations may represent the same point in reality. Therefore, an approximation is necessary when using point clouds to match model objects. Furthermore, the geometry of BIM objects is commonly described as meshes, with triangles and polygons representing object surfaces. Checking if a point lies within a surface of an object is too computation intensive considering a camera stream of thousands of points within each frame. Octrees are the ideal data structure for analysing occupancy of spaces that reflect real world environments. An octree is an efficient representation of occupied space as it recursively subdivides space into eight cubes that may either be occupied or free. The cubes are ordered in a tree data structure which allows quick lookup and accelerates lookup times for larger occupied areas.

Octrees are also the basis for the UAV’s representation of navigable space. Octomap [45] dynamically manages a representation of occupied space in the UAV’s environment, which is needed for mapping of and navigation in environments that are being explored. Pre-loading the octomap with the known occupied space of the building model has a positive effect on the UAV’s capability of finding a shortest viable path, since all known structures are automatically included in the path planning of the local planner.

In order to accurately register the incoming point clouds with the existing environment representation, the current position and orientation of the UAV is required. Since the PX4 autopilot constantly localises itself in space by fusing all sensory inputs in a state estimator, each incoming point cloud can be associated with the UAV’s current position. The mavros node publishes each newly perceived pose in space together with a timestamp of the running ROS session. Registering a point cloud is ideally done by associating a point cloud with the pose measured at the time of creation of the source images. Despite being fast enough, the computation of disparity maps takes a small amount of time. Therefore it is important to preserve the timestamp of each frame of the stereo camera to find the matching pose of the UAV to associate with with generated point cloud.

**Figure 5:**
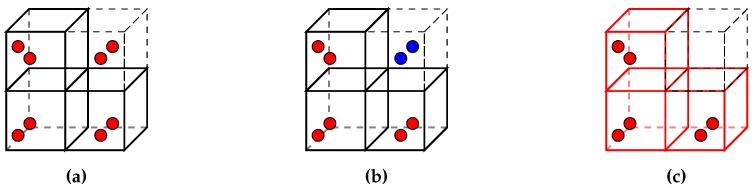
Simplified illustration of the point cloud subtractor using the octree to subtract points that do not belong to the as-planned representation of an inspection object. (**a**) Input point cloud (red points) aligned with voxels of as-planned representation (solid boxes). (**b**) Elimination process: discard points that do not match an octree leaf (blue points). (**c**) Result: matched octree leafs are marked occupied (red).

Figure 5a shows the initial status of data as it arrives in the node. The existing structures are displayed as boxes with thick solid outlines and the input data is illustrated by red points. The boxes represent leafs of the octree, which is an efficient discretisation of the building geometry. Some points are placed within a box of the octree, others are outside the octree. Since the octree is a discretised representation of the as-planned model, all points inside the octree shall be discarded. The goal is to efficiently identify all points, that lie outside the octree. The point cloud subtractor makes use of the PCL data structure OctreePointCloud and its function isVoxelOccupiedAtPoint to test each input point within in the octree and keep a set of outlier points, marked as blue points in Figure 5b,c.

The result of this operation is a point cloud frame that represents only objects that are new or modified compared to the previous model of as-built or as-planned status. Furthermore, the octree leafs are now marked occupied where points of the physical object were registered.

Figure 4 illustrates an exemplary subtractor operation. The input data of the point cloud subtractor consists of a point cloud frame and the as-planned octree which is derived from the BIM. In this case, the as-planned octree represents the upper part of the wall, seen in the input point cloud. After the subtraction operation, the point cloud only shows points that belong to the target object. The point cloud has semantic value and can be attributed to a specific BIM object. The number of occupied cells in the matched octree represents the degree to which the target object is matched. The matched octree is updated continuously with each incoming point cloud frame. Provided with point clouds that cover the whole surface of the target object, all cells in the matched octree should be updated. The matched octree shown in Figure 4 exhibits some holes in parts of the structure that were not yet covered by incoming point clouds. The matched octree completes the concept of detecting the presence of physical objects for the verification of process finalisation.

The subtracted point clouds are transferred to the point cloud assembler.

### 3.4. The Point Cloud Assembler

The point cloud assembler (see pc_assembler in Figure 2 and Figure 4) has the purpose of creating a complete point cloud representation of a target object. This accumulated point cloud is meant for storing a visual as-built representation of the reconstructed object and is not relevant to the presence detection described in Section 3.3.

During each flight, the UAV captures extensive amounts of point cloud data. Subsequent point cloud messages are assembled at the end of the toolchain with the intention to create complete views on objects from all perspectives. Although the input rate can be throttled to a value lower than that of the navigation system, it must be fast enough to ensure that no objects are overlooked while the UAV is moving. The information of single frames, accumulated intervals of frames or even whole flight sequences is too large to make sense to a human operator when simply displayed on a screen.

Each point cloud frame, regardless of it being generated by a TLS or a flying stereo camera, can only represent points visible from the location of the sensor. For 3D mesh reconstruction purposes, point clouds of objects should be complete. In order to create complete point clouds of physical objects, point clouds must be created from multiple perspectives and then these frames must be aligned and joined. The point cloud assembler (pc_assembler) collects point cloud frames and their respective transforms, a ROS description of pose, orientation and the scale of objects. It joins multiple frames and its output is a single, accumulated point cloud.

Whereas the localisation error found in single frames can be accounted for, the accumulation of multiple point clouds also accumulates the errors of each frame. Furthermore, the overlapping sections of point cloud frames accumulate points for the same parts of surfaces. These issues can be addressed in different ways. The toolchain only accepts point clouds that were captured when the UAV’s current motion was low. Acceleration values present in the mavros interface provide the necessary information. Furthermore, the statistical outlier removal and VoxelGrid filter found in PCL help maintaining an assembled point cloud. The statistical outlier removal effectively removes points that are not needed. The VoxelGrid filter periodically downsamples the point cloud to remove duplicate points in similar locations, also keeping the point cloud at a computation friendly size. An evaluation of the point cloud assembler is not in the scope of this work which has the primary goal of automatically detecting the presence and finalisation state of BIM objects.

The last step is the pcd_export node, which receives point cloud messages and writes artefact files in the common PCD file type.

### 3.5. The Inspection Planner

Apart from processing visual information and checking the status of as-built objects, the toolchain manages the UAV’s flight tasks. The autonomous capabilities of PX4 coupled with avoidance functionality, relevant to this use case, may be summarised as follows:

1. Fly towards target positions determined by geocoordinates.
2. Avoid obstacles that appear on the way.
3. Repeat 1 and 2 for waypoint mission behaviour.

Being able to dynamically detect and avoid obstacles is the most important aspect for automated and safe operation of UAVs in applications like construction inspection. However, one other crucial aspect needs to be considered for this application. The UAV’s navigation space is empty and is only being fed information about occupied space during flight. With no knowledge about its environment, the UAV’s path planning unit cannot make informed decisions about how to reach its target locations. Given a location as navigation target, the UAV would start exploring its environment with a general orientation towards the target. If it encounters a blocked path, for example building structures in between, it changes its course and attempts to circumvent the obstacle. Without knowing whether circumventing an obstacle to the left, to the right or flying over it leads to the ideal path, the UAV needs substantial exploration time before even reaching its target. The inspection planner node fills this gap by utilising the georeferenced BIM data as a priori knowledge. Based on the octree derived from BIM data, the inspection planner generates complete inspection missions. Missions include short paths towards the inspection object, circling around the object to gather complete visual information and the way back towards a designated landing position. The waypoints of the inspection pattern are generated as follows:

1. Create an object-aligned bounding box around the inspection object.
2. Reduce the bounding box to an area of four vectors at half the height of the former box.
3. Expand the area by a pre-defined distance in each lateral dimension.
4. Place waypoints on each edge of the expanded area, with a pre-defined distance between waypoints.

The distance parameters in steps 3 and 4 should be chosen according to the camera’s properties. Waypoints placed too far away from the object or from each other may result in incomplete coverage of the object, depending on the camera’s resolution and field of view. This is considering that the UAV can only point its stereo camera towards the inspection object while halting at waypoints. While reaching for the next waypoint, the UAV must point its stereo camera in forward flying direction. This limitation may be avoided by installing a second depth camera on the UAV that points sideways. Since the inspection planner always generates waypoints around target objects in clockwise manner, the second depth camera must point to the right.

## 4. Evaluation

The proposed toolchain was evaluated with the same simulation environment it is developed in. The simulation environment is an ideal tool to assess the individual components of the presented method. The ability to control, repeat and compare test runs is a necessary prerequisite for finding the right sets of parameters and locating error sources in case of unexpected results. The intention behind the following evaluation is to demonstrate the feasibility of the proposed method and identify technical capabilities and limitations of the implementation. The main emphasis of the method is automation and therefore the evaluation is carried out solely on the presented software components with no human intervention required. The only exception is the visual preparation of the simulation environment as discussed in Section 3.1. Flight tests on real buildings will reveal further insight about the method but are not within the scope of this work. After consolidating the general feasibility of the proposed approach, an evaluation on hardware will be carried out.

It is expected that, apart from mandatory calibration of the flight control, no further work is required to execute the toolchain on a UAV with a sufficiently powerful companion computer and integrated stereo vision hardware. Intel Aero satisfies all requirements of the toolchain and should therefore be mentioned as a suitable hardware platform. Aero includes a PX4 compatible flight control and a depth camera, which replaces the software-based disparity computation of the stereo_image_proc node. An essential aspect to consider is the on-board companion computer that is responsible for both obstacle avoidance and analysis of structural elements. Aero’s companion computer is equipped with an Intel Atom processor with a maximum frequency of 2.56 GHz on four native threads. The complete toolchain, including the software-based disparity computation and 3D visualisation of the simulation scene, was built and tested on a Celeron CPU (2.5 GHz and four native threads) and showed no performance issues. Considering that the execution on the UAV does not require CPU time for disparity computation, visualisation and execution of the SITL firmware, performance is sufficient.

The simulation-based evaluation involves some data preparation which shall be described before going into detail about the observations and adjustments that were made during the evaluation.

### 4.1. Setting up Simulation Experiments

**Figure 6:**
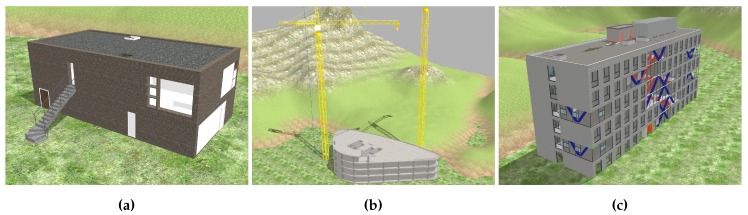
Three simulation scenes with construction states derived from BIM. (**a**) Duplex apartment building. (**b**) Mefisto construction model. (**c**) Hochschule Bochum (HSBO) building model.

In order to evaluate a flight mission for analysing the state of a construction site, a virtual as-built state of the construction site needs to be constructed. Furthermore, the simulation of vision-based autonomous flight in ROS requires visual information as input for the cameras. The Gazebo simulation application works with world descriptions that provide realistic robot environments with complex mesh data, lighting, collisions and physical aspects like friction and gravity. The building data for the as-built representation is imported in the form of 3D asset files. Common compatible asset formats include Collada, STL and OBJ. Apart from STL, which only describes geometry, all asset formats also include information about surface materials, textures and shading, which are important when creating a realistic environment for visual navigation. Exemplary BIM projects were embedded in realistic scenes to represent the real state of the construction site for each experiment. Figure 6 illustrates the three construction scenes that were created for development and evaluation purposes. The as-built states were derived from IFC files and converted to Collada format. The conversion process is accomplished with a conversion application based on xBim [42]. Initial tests on converted building models yielded poor disparity maps due to the plain surfaces of the Collada exports.

The creation of disparity maps and further point clouds in simulation is based on semi-global matching (SGM) [44]. SGM and its block matching variant rely on visual features in image pairs to identify matching pixels or blocks. Parts of images that have little distinctive visual features are known to cause wrong disparities [44]. This is a crucial aspect when generating disparity maps from synthetic video feeds in the simulation. Scene objects with no textures or other surface properties cause erroneous disparity maps and therefore bad distance readings for the obstacle avoidance system. The quality of the reconstructed point clouds also determines the ability of the subtractor node to correctly match perceived geometry with the model’s properties. The material properties of IFC objects are represented by coloured surfaces but lack a realistic visual representations in the form of textures. The quality of disparity maps generated from synthetic data was greatly improved by adding photo-realistic textures of typical construction materials to facade elements. This is a limitation that only applies to the simulation. Camera input from physical objects under natural lighting exposes more visual features than a simulation.

**Figure 7:**
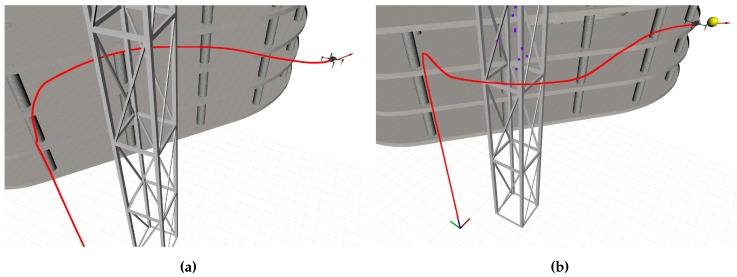
Two flight trajectories (red line) of the UAV avoiding collision with a crane soon after takeoff. (**a**) The trajectory shows a clockwise motion around the crane. (**b**) In another flight the crane was circumvented in a counter-clockwise motion.

Figure 7 shows visualisations of two recorded flights with an obstacle in the desired flight path of the UAV. The flight trajectory is represented by a red line. The UAV was given a flight mission with first task being vertical takeoff and the second task being a waypoint a few metres away. After takeoff the UAV would begin flying towards the waypoint in straight forward flight but the obstacle avoidance system recognised the girders of the crane. Next, the local planner starts creating a flight path along the unoccupied parts of the navigable space, resulting in a curve around the object. While circumventing the object, the local planner visibly tries to direct the UAV back on the original flight path. As soon as there is no object block the path towards the waypoint, the local planner lets the UAV continue on its straight path to the target.

**Figure 8:**
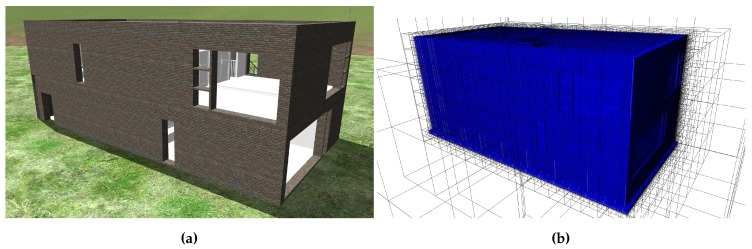
The octree of the building model at present state is used as a computationally efficient representation of navigation space and for the comparison of model geometry vs. reconstructed geometry. (**a**) A BIM with additional textures rendered in Gazebo for a realistic representation of the as-built state. (**b**) The corresponding octree for navigation and point cloud processing represents the as-planned state.

In the simulation-based evaluation the exemplary BIM projects serve two purposes, one being the representation of the as-built state, the other being the source for deriving the as-planned state. Internally, the as-planned state is managed in the form of an octree for efficient analysis of spatial occupation between model and point clouds. Figure 8 shows an example of a simulation representation of a BIM derived from IFC and its corresponding octree representation. In contrast to the manual creation of the simulation-specific 3D scenes, the as-planned representation is created completely automatic. A script in the toolchain invokes binvox [46,47] for voxelisation of the model data and binvox2bt [45] for creating the octree of the voxel data.

The preparation of experiments required making structural changes to BIMs as they would occur between states of a 4D-BIM and exporting these states as individual IFC artefacts. With different states at hand, it is then possible to set up two different types of experiments. One type describes a scenario in which the as-is and as-planned representation are identical. In this scenario, the UAV should reliably detect the inspection object. The other scenario is built with an as-is representation that deviates from as-planned. The real world equivalent of this test is a due date for a specific structural element which is not present at the time of inspection. In this scenario the as-is representation lacks the inspection object and the UAV is expected to conclude that this specific object is not present in the as-is scene.

### 4.2. Case Study

The proposed method was evaluated in tests on three different BIMs. The tests revealed practical insights of the toolchain’s capabilities and limitations. Each of the tests was conducted with the complete toolchain to demonstrate that all presented components work together and not just individually. Apart from the manual preparation of two visual simulation scenes per test, described in Section 4.1, each test finished fully automatic by the following procedure:

1. Parse the BIM artefact with new structures and generate an octree of the geometric extent of the structures.
2. Generate the flight mission in geodetic coordinates that guides the UAV around the target region.
3. Initialise the navigation space for the UAV with known structures as pre-loaded environment knowledge for the local planner node.
4. Switch to Mission flight mode with obstacle avoidance enabled and arm the UAV.
5. During flight the UAV captures point cloud data of objects in the target region with registration of each frame of points according to the UAV’s current position.
6. Points are continuously accumulated in a second octree that resembles the reconstructed structure.
7. Each new measurement is processed in the subtractor node.
8. After finishing the flight mission, the two octrees, model and reconstruction, can be compared.

The toolchain’s functionality and performance were evaluated in two simulated test scenarios with three experiments each. These experiments should provide an understanding of whether the proposed method is able to discern individual structural elements and produce a numerical statement about an inspection object being present or missing. True negatives were not tested because the subtractor is implemented to only consider points that lie inside the space of the as-planned object. Therefore, all other points (true negatives) are automatically discarded.

**Figure 9:**
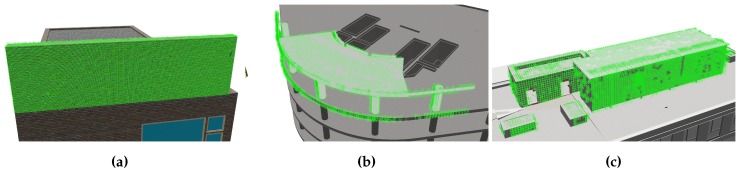
Results of true positive tests (scenario 1). Structural objects are indicated by the schedule and fully present in the as-built scenes. Green annotations show octree leafs that were correctly matched during flight. (**a**) a single wall in scene 1 (94.8%). (**b**) multiple columns and a slab in scene 2 (76.53%). (**c**) a complete structure on a roof in scene 3 (82.16%).

The first scenario is a true positive test for each of the three scenes. Figure 9 shows a visualisation of the three experiments conducted in this scenario. True positive in this context means that as-is and as-planned are identical and therefore the matched structures of the subtractor node are correctly classified. The subtractor matches leafs of the as-planned octree as occupied if points were detected in the leaf’s space. Therefore, the quality of a matching procedure can be assessed by counting the number of occupied leafs of the reconstructed octree in relation to the number of leafs in the as-planned octree. An ideal matching is defined as a reconstruction octree having the the exact same leafs marked occupied as the as-planned version. Realistically though, the geometry reconstruction has a certain error. The goal of this scenario is to show if this error affects the matching procedure.

Experiment 1 (see Figure 9a) resulted in 94.8% correctly matched octree leafs. Visual observation revealed that the disparity maps, the basis of the point clouds, were very accurate. This is probably due to two reasons. First, the inspection object is of little geometric complexity. With the camera being pointed almost perpendicularly towards the object’s surface, large areas of each image pair can be identified as connected. In contrast, curved surfaces are harder to reconstruct for the stereo image processor. A second factor might be the photo-realistic brick texture applied to the surface, which is also beneficial to the generation of the disparity maps. The missing 5.2% can be explained by occlusion. The inspection object, a wall, is seated on another wall. There is no way for the cameras to capture visual information about the downside of the object.

Experiment 2 (see Figure 9b) yielded a correct matching of 76.53%. As mentioned above, the accuracy of disparity maps degrades with curved surfaces and surface not perpendicular with the camera’s view. On visual inspection the curved sections of the object revealed higher inaccuracies than other surfaces. Another section of inaccuracy is the large slab on top of the object. There are visible spots with missing green annotations, which were not matched during the procedure. The inspection planner currently generates flight paths that guide the UAV around inspection objects, making visual coverage of larger horizontal areas more difficult. This problem can be countered with a path generation procedure that also considers flight paths above objects above a certain lateral extent.

Experiment 3 (see Figure 9c) came out at 82.16% correctly matched volume. Visual inspection of the reconstructed octree shows that occlusion and horizontal surfaces were, again the main reason for unmatched surface areas.

Concluding the evaluation of scenario 1, all objects were detected with true positive matching scores greater than 75%. The experiments exposed problems with inaccuracies, however, the quality of reconstruction seems to improve with more realistic surface properties.

**Figure 10:**
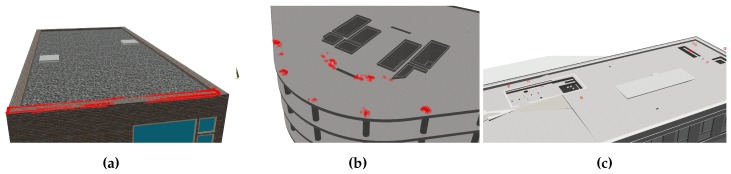
Results of false positive tests (scenario 2). Objects from Figure 9 are required by the schedule but missing in the as-built scenes. Red annotations show parts of the inspection object being matched erroneously. (**a**) Several false positives occurred closely above the top face of another wall object (12.74%). (**b**) Some false positives above neighbouring columns (2.32%). (**c**)Few false positives with seemingly random distribution (0.12%).

The second scenario tested for false positives. The detection procedure should not only positively identify objects that are present (scenario 1), it should also conclude reliably that a specific object is not present when it is in fact missing. Figure 10 shows a visualisation of the three experiments of scenario 2. The experiments are identical to the ones of scenario 1, the only difference being that the inspection objects from as-planned are missing in the as-is representation of the simulation. Each experiment should ideally finish with 0% matched volume. Realistically though, the point cloud-based matching procedure receives erroneous points with inaccurate locations. Such inaccuracies mean uncertainty. Therefore, the goal of scenario 2 is to ascertain whether the degree of uncertainty is low enough to reliably detect missing objects.

Experiment 4 (see Figure 10a) resulted in 12.74% falsely matched volume, the highest number among the measurements taken in scenario 2. There distribution of red annotations, denoting falsely matched octree leafs, shows that all wrong measurements were taken at the bottom part of the missing object. This is caused by inaccurate points that were captured from the visible upper surface of the neighbouring wall. Some points reconstructed from that surface fell into the space of the search volume and triggered false matches. A possible countermeasure to this phenomenon could be to exclude border regions between the inspection object and other connected objects from the matching procedure.

Experiment 5 (see Figure 10b) produced a falsely matched volume of 2.32% the size of the search volume. Upon visual inspection, a majority of the points triggering false matches occurred on top of column elements from the storey beneath the inspection object. The 3D geometry of the model exposed Z-fighting in those locations. It is a phenomenon where congruent faces of 3D meshes cause rendering engines show flickering surfaces. Each subsequent image pair from the stereo camera therefore showed different wrong visual representations in these locations and therefore the point cloud reconstruction was affected.

Experiment 6 (see Figure 10c) resulted in 0.12% falsely matched volume. In this experiment, the search volume was cropped in the border region at the bottom, to avoid the problem observed in experiment 4. There are only few false positives with random distribution.

Concluding the experiments of scenario 2, the matching performance shows sufficient accuracy in detecting missing objects.

The tests for true positives and false positives show that, considering error thresholds, the automated inspection system is able to determine whether single BIM objects are present or missing in the UAV’s environment (see Table 1). The tests also revealed some limitations of the system, with occlusion being the most notable. As shown in the experiments, the system is only intended for use on exterior building structures but occlusions cannot be ruled out and single objects may be encompassed by others. If a majority of an object’s surface is occluded, the performance of the point cloud reconstruction degrades significantly. This is a systemic limitation of all vision and camera-based approaches to as-built generation. However, this limitation only applies to the data processing aspect of the presented approach. The concept of automated data acquisition with UAVs is not affected by this limitation as it is independent of the processing procedure. Regarding the acquisition method, UAVs offer the highest potential for maximising visual coverage as they can obtain virtually any vantage point.

The inspection planner requires setting two parameters: the distance between UAV and object surface and the distance between waypoints (see Section 3.5). These parameters, along with the depth of the octrees, were determined by the following considerations.

The distance between UAV and object surface (step 3) not only determines the coverage of the stereo camera but also the resolution in points per cm on the surface. In the case study, a distance of 3.5 m was used, along with a Field of View (FoV) of 80° and a resolution of 720×720 points. These parameters determine a resolution of 1.22 points per cm. For comparison, the horizontal FoV Intel Realsense D435 is 87° at an output resolution of up to 1280×720 points, determining a maximum resolution of 2.03 points per cm at the given distance. In regard to checking octree cells, the resolution should allow for every cell of the octree to be matched by at least one point of the point cloud. The octrees in all experiments were generated with a depth level of 16. The resulting octree resolutions (size of the smallest octants) used in the experiments are in the range of ∼6.5 cm to ∼24 cm. Considering object sizes in the range of 8 m–30 m, these resolutions were found to be of sufficient detail to describe the target object’s shape. With an octree depth set to 16, the resolution of the octree increases proportionally to object size. In order to maintain the ability to match every octant with a point cloud, the minimum octant size should not fall short of the expected point cloud resolution. This set of parameters is suitable for objects greater than 1 m. For objects smaller than that, either the distance of the UAV should be reduced or a lower octree depth may be applied.

## 5. Conclusions and Outlook

This work is a study on automating and improving the process of as-built data generation. Construction management requires more information on the as-built state at increasing rates in order to make informed decisions. Only a fully automated method can effectively satisfy this demand. The framework employs autonomous UAVs as a utility for fully automatic acquisition of ordered and meaningful information on the state of structural objects. The prototype of the proposed framework is capable of guiding a UAV around building structures, effectively enabling it to capture as-built information on an object level. The vision-based collision avoidance functionality of the UAV ensures safe and efficient operation, without interfering with ongoing construction work. The self-localising UAV significantly improves the process, as scanned point clouds can inherit the UAV’s position information for each frame. Therefore, no additional point cloud registration is required. Furthermore, the toolchain’s ability to eliminate all points of known or unmodified objects greatly improves the value of the as-built data. Despite being under active development, the open source autopilot software and the Robot Operating System (ROS) provide a solid foundation for practical applications of autonomous UAVs. The main focus of this work is employing UAVs for capturing as-built data. As referred to in the Section State of Technology, there are many approaches of analysis and decision-making that rely on structured and repeatable data acquisition. This work aims to provide a framework for efficiently capturing the input data for all kinds of analysis purposes that require structured and repeatable as-built information. The presented approach for segmenting point clouds and detecting distinct objects shows that these expectations are met by the autonomous UAV and the software that controls it. This is a work in progress. Following the simulation-based tests, the toolchain will be evaluated on real hardware and in practical case studies. The isolated groups of new or modified objects will be used for automated progress monitoring of processes in 4D-BIM.

The proposed approach could be improved in the following ways: the application sources its information from BIM data. After finishing an inspection flight, results should be persisted in IFC files that include all detected objects. The geometry of objects should be imported from the as-planned model and linked to the captured as-built data, stored as point cloud data and images.

The fixed depth of octrees used in the case study determines that octants increase or decrease in size proportionally to object size. Octant size and point cloud resolution directly determine the quality of the presence detection. The robustness of the presence detection could possibly be improved by optimising the desired octant size and computing the required octree depth for each object.

Furthermore, the case study showed that ignoring border regions to surfaces of adjacent objects reduce the number of false positives in the matching procedure. This improvement could be added to the automatic octree generation.

## Figures and Tables

**Table 1:** Comparison of octree volumes for each evaluation case.

Volume	As-Planned (m^3^)	True Positive (m^3^)	False Positive (m^3^)	True Positive (%)	False Positive (%)
duplex	3.65	3.46	0.47	94.80	12.74
mefisto	98.80	75.61	2.29	76.53	2.32
hsbo	121.48	99.80	0.15	82.16	0.12
